# Head to Knee: Cranial Neural Crest-Derived Cells as Promising Candidates for Human Cartilage Repair

**DOI:** 10.1155/2019/9310318

**Published:** 2019-01-15

**Authors:** Ihsène Taïhi, Ali Nassif, Juliane Isaac, Benjamin Philippe Fournier, François Ferré

**Affiliations:** ^1^Laboratory of Molecular Oral Pathophysiology, INSERM UMRS 1138, Cordeliers Research Center, Universites of Paris-Descartes, Pierre and Marie Curie and Paris-Diderot, 15 rue de l'école de médecine, Paris, France; ^2^AP-HP, Charles Foix-Pitié Salpêtrière Hospital, Dental Department, Ivry, France; ^3^AP-HP, Bretonneau Hospital, Dental Department, Paris 75018, France; ^4^Mondor Institute for Biomedical Research (IMRB), INSERM U955, Team 5, Faculty of Medicine, University of Paris-EST, Créteil, France; ^5^Laboratory of Pharmacology of Cerebral Circulation, EA4475, Faculty of Pharmacy, University of Paris Descartes, France; ^6^Reference Center for Dental Rare Diseases, Rothschild Hospital, Paris, France

## Abstract

A large array of therapeutic procedures is available to treat cartilage disorders caused by trauma or inflammatory disease. Most are invasive and may result in treatment failure or development of osteoarthritis due to extensive cartilage damage from repeated surgery. Despite encouraging results of early cell therapy trials that used chondrocytes collected during arthroscopic surgery, these approaches have serious disadvantages, including morbidity associated with cell harvesting and low predictive clinical outcomes. To overcome these limitations, adult stem cells derived from bone marrow and subsequently from other tissues are now considered as preferred sources of cells for cartilage regeneration. Moreover, with new evidence showing that the choice of cell source is one of the most important factors for successful cell therapy, there is growing interest in neural crest-derived cells in both the research and clinical communities. Neural crest-derived cells such as nasal chondrocytes and oral stem cells that exhibit chondrocyte-like properties seem particularly promising in cartilage repair. Here, we review the types of cells currently available for cartilage cell therapy, including articular chondrocytes and various mesenchymal stem cells, and then highlight recent developments in the use of neural crest-derived chondrocytes and oral stem cells for repair of cartilage lesions.

## 1. Introduction

Cartilage tissue is a constituent of many structures of the human body such as the nose, the articular discs, and the synovial joints. In the latter, hyaline cartilage covering the extremities of bones prevents them from rubbing together to ensure joint mobility and distributes the biomechanical forces to the underlying subchondral bone. Articular discs are composed of a more rigid cartilage tissue, fibrocartilage, with a denser organization of collagen fibers within the cartilage matrix, endowing them with shock absorption properties. A few structures, mainly in the external ear and larynx, contain a different, very flexible type of cartilage composed of numerous elastic fibers [1].

The load-bearing properties of the articular cartilage are correlated to the activities it performs and its location in the body. Hyaline cartilage is a specific type that contains an extracellular matrix (ECM) rich in proteoglycans (notably aggrecan) and collagen (mostly type II, but also types V, VI, IX, X, XI, XII, and XIV). The linear polymers keratan sulfate and chondroitin sulfate, which are carboxyl and sulfated glycosaminoglycans, carry negative charges that confer a high affinity for water and thus contribute to the viscoelastic properties of the articular cartilage [2]. Chondrocytes comprise 2% of the total volume of articular cartilage. They produce the cartilage matrix and maintain homeostasis in the diverse articular zones. Each zone (superficial or “lamina splendens,” intermediate zone, deep zone, and calcified layer) features a specific molecular composition and architectural organization. Alteration of any zone (e.g., by injury) may lead to degeneration of the articular cartilage. The effects depend on the severity and depth of the injury. Due to the absence of blood vessels [3], superficial- and partial-thickness defects do not elicit fibrin clot formation. Defects induce local inflammation, chondrocyte proliferation, and matrix synthesis, but these reparative processes cannot restore the surface of the cartilage. Moreover, in the neomatrix, the collagen network is disorganized and the quantity of proteoglycans is lower, which favors the hydration of the matrix [3–6]. All these aspects of cartilage healing result in decreased stiffness and increased transmission of forces to the subchondral bone. When a defect extends through the entire cartilage to the subchondral bone, blood clots first fill the defective areas and endogenous stem cells are activated. Granulation tissue is substituted by a fibrocartilage that exhibits less effective mechanical and biological properties than the hyaline cartilage.

Articular cartilage defects arising either from acute traumatic injuries or from chronic inflammatory diseases like osteoarthritis (OA) are disabling health problems that affect both young and old persons worldwide. These defects are associated with pain and loss of joint mobility, and they impact the quality of life, including physical, social, and economic well-being. Lesions involving both the articular surface and discs may be caused by diverse etiologies. Many defects are initiated by trauma and affect younger adults; in such patients, the aim of the treatment is to preserve the integrity of the joint and its functions [7]. For critical size defects or disabling lesions, the ultimate treatment consists of invasive surgical procedures to replace the articular surface by a prosthesis or arthroplasty. In the United States, more than 300,000 arthroplasty procedures are performed each year to replace the femoral head in the hip articulation [1]. It is estimated that by 2050, nearly 3.5 million primary total knee arthroplasties and 600,000 primary total hip arthroplasties will be performed annually in the USA [8]. The limited lifespans of these prostheses make them suitable only for older patients, not for young ones. This emphasizes the need for novel, effective therapeutic strategies for cartilage defects, especially in young people, to avoid extensive cartilage damage or repeated arthroplasty surgeries.

**Figure 1:**
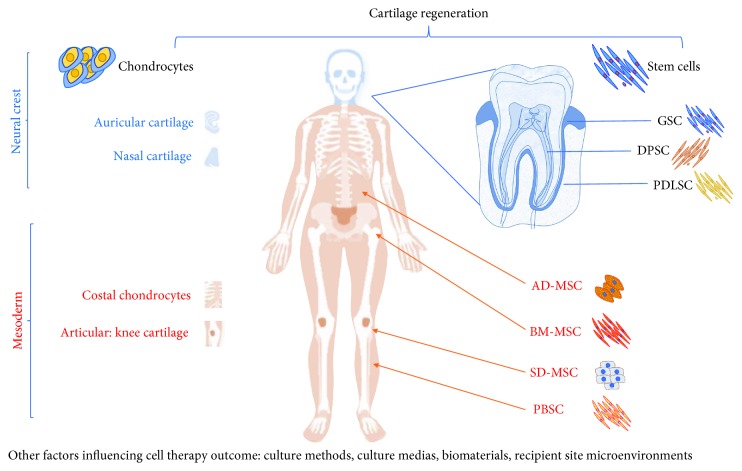
Cell therapy for cartilage regeneration. Principal sources of cells used for cartilage repair according to their embryonic origin.

Confronting all the difficulties encountered and the failures surrounding the surgical procedures, cell-based therapies for cartilage repair began to be conceptualized during the past two decades. After some promising early results suggesting that cell therapies might be quickly adapted for cartilage regeneration, ensuing difficulties have impeded this approach. One important limitation was found to be the cell source. Indeed, it is now clear that the processes of cell commitment, differentiation, amplification, and immunomodulation are in large part linked to the origin of the implanted cells [9–11]. Here, after first reviewing the different cell sources currently being used for cartilage repair (Figure 1), we focus on neural crest-derived cells, discussing their structural and functional features and their therapeutic application to articular defects.

## 2. Mesoderm-Derived Cells for Cartilage Repair

### 2.1. Mesodermal Chondrocytes

Autologous chondrocyte implantation (ACI) was first used in a clinical trial in 1994 by Brittberg et al., who transplanted autologous articular chondrocytes into the femoral head of 16 patients and the patella of 7 others, achieving excellent to good results in most cases (14 of the 16 patients with femoral head treatment). The transplants resulted in the formation of neocartilage, with hyaline cartilage appearance in 11 of the 16 femoral head transplants and 1 of the 7 patellar cases [12]. However, despite encouraging early results, the use of articular chondrocytes had many drawbacks, including morbidity linked to cell harvesting and low predictive clinical outcomes [13].

Novel sources of chondrocytes include costal [14], auricular [15], and nasal cartilage [16] (developed later) and, as recently reported, allogenic chondrocytes with no history of joint disease [17]. Matrix-assisted autologous chondrocyte transplantation (MACT) is another emergent cell-based treatment. It was introduced to improve the ACI procedure and consists of transplanting chondrocytes within a biomaterial scaffold. These hybrid biomaterials are fixed into the cartilage defect with a fibrin glue. This strategy showed favorable mid- to long-term clinical outcomes for knee cartilage treatment [18]. Many clinical trials have been performed, and the method was recently approved by the US Food and Drug Administration [19]. The main drawback of the ACI+MACT approach is that it is a two-step procedure. Thus, it requires two surgical sites [20]. Moreover, transplantation of insufficient numbers of chondrocytes and their limited proliferative ability may be limiting factors for cartilage regeneration (the question of using scaffolds to promote cell implantation is not discussed further in this review).

It is also important to consider the unpredictability of the chondrogenic potential of the transplanted cells, which may be more problematic when nonchondrocytes are used in cartilage cell therapy. Indeed, when transplanted *in vivo* after harvesting or when cultured *in vitro* in monolayers, the cells may dedifferentiate to express a fibroblast-like phenotype, which may result in reduced collagen type II and proteoglycan synthesis [21]. Moreover, the quality of the cultured chondrocytes greatly affects the outcome of cell therapy [22]. Likewise, the gene expression profile in the microenvironment of a cartilage lesion seems to be an influencing factor of the early outcome after ACI. Indeed, elevated expression of inflammatory cytokines like interleukin 1*β* (IL-1*β*) and vascular endothelial growth factor receptor 1 (FLT-1) is associated with early graft-related adverse effects [23]. Furthermore, IL-1*β* expression is correlated with negative clinical outcomes over the long term [24]. The limitations of using the articular chondrocytes prompted searches for new sources of cells, and adult stem cells derived from bone marrow [25] were quickly regarded as a good alternative [26].

### 2.2. Mesodermal Mesenchymal Stem Cells

Mesenchymal stem cells (MSCs) were first reported in 1968 by Friedenstein et al., who observed that a fraction of adherent cells from bone marrow aspirate was able to self-renew and displayed colony-forming capacity. These cells were later demonstrated to be able to differentiate under suitable conditions into osteoblastic cells, adipocytes, or chondrocytes [25, 27]. In bone, MSCs are essential components of the hematopoietic stem cell microenvironment because they participate in hematopoietic cell turnover [28–30]. Initially discovered in bone marrow, these cells can also be isolated from various other tissues (adipose tissue, synovial membrane, umbilical cord, and so on). They have been localized especially around blood vessels (pericytes), and they participate in tissue homeostasis and healing after injury by modulating the contributions of other cells involved in the inflammatory/repair response [28, 31]. Currently, MSCs are the most commonly used stem cells in human therapy and regenerative medicine. Moreover, their ability to undergo chondrogenic differentiation makes them a promising cell type for repair of cartilage defects [26].

Mesodermal MSCs have been introduced into cartilage regeneration therapies in conjunction with biomaterial matrices and chondrogenic differentiation initiated and tightly regulated by growth factors, predominantly from the TGF-*β* superfamily [32]. Many animal and clinical studies have been conducted during the past 17 years. Wakitani et al. in 2002 [33] were the first to surgically implant bone marrow-derived MSC (BM-MSC) embedded in a collagen gel into osteoarthritic knees. They observed higher arthroscopic and histologic scores compared to a control patient group. Their use is considered safe and shows clinical improvement and satisfactory magnetic resonance imaging (MRI) and macroscopic results. However, histologic results remain controversial [20, 34].

BM-MSCs remain the most commonly used cell type in cartilage regenerative medicine. Their use in cartilage regeneration is supported by many *in vivo* and clinical studies [34, 35]. BM-MSCs were found to be at least as effective as autologous chondrocytes in terms of cartilage regeneration [35].

However, due to the pain caused by BM-MSC harvesting, other sources of MSC have been explored. Adipose-derived stem cells (AD-MSCs) are another convenient type of MSC because they can be easily isolated from excess human adipose tissue by liposuction. Their use for chondrogenic differentiation is mainly based on the isolation of the stromal vascular fraction (SVF) after enzymatic digestion of the adipose tissue. They are used in human clinical trials for articular cartilage regeneration [36]. Despite their abundance and ready availability, AD-MSCs have lower chondrogenic potential than BM-MSC [37], especially when the cells are harvested from older patients [38].

MSCs from other sources were recently isolated and showed higher potential for chondrogenic differentiation under suitable conditions. Synovial membrane-derived mesenchymal stem cells (SD-MSCs) showed higher chondrogenic potential than BM-MSC *in vitro* [39]. *In vivo*, the synovial space is the main source of nutrients and the site of gas exchange (via the synovial fluid) and the synovial membrane produces prechondrocytes which give rise to stem cells involved in cartilage repair after minor trauma [40]. Sekiya et al. transplanted SD-MSC into 10 femoral condyles of patients with symptomatic single cartilage lesions and had satisfactory outcomes after 3 years of follow-up [40].

Peripheral blood stem cells (PBSC), which are isolated from simple venous samples, have similarly been used for cartilage regeneration in human patients, first by Saw et al. in 2011 [41]. In another human clinical trial, the same authors introduced PBSC into chondral knee lesions by using subchondral drilling and had encouraging histological findings, with neocartilage tissue formation [42].

Wharton's jelly stem cells (WJSC) have also been proposed as an alternative to BM-MSC and AD-MSC [43]. Their advantages include painless harvesting from the human umbilical cord, a high yield of stem cells, and multilineage potential. The absence of ethical concerns when compared to embryonic stem cells (ESC) has made them a promising tool for the treatment of cartilage defects. *In vitro*, they show a better expression of chondrogenic differentiation-related genes (SOX9, Col2A1, ACAN, and COMP) than BM-MSC [43]. They have also been used in intravenous transfusion in one patient with aggressive multiple sclerosis, with favorable outcomes after four years of follow-up [44]. In summary, most long-term clinical data relate to the use of BM-MSC. Like chondrocytes obtained in the MACT and ACI techniques, BM-MSCs are difficult to obtain and their harvesting is associated with a risk of morbidity. AD-MSCs have lower chondrogenic potential compared to SD-MSC, PBSC, and WJSC.

The difficulties associated with collecting BM-MSC from donors as well as their *in vitro* properties (proliferation, chondrogenic differentiation) make them less attractive for cartilage repair than in the past. An important drawback in inducing a standardized functional neocartilage using MSC of the mesodermal origin concerns the differences in chondrogenic potential related to the source of the MSC, as pointed out by Mehlhorn et al. [45]. Indeed, cartilage ECM was found to be different between AD-MSC and BM-MSC transplants. Furthermore, patient-to-patient variation also impacts the outcome of MSC cartilage regeneration, especially in older patients, in which the chondrogenic potential of MSC is reduced. Maintenance of the chondrocyte phenotype from a differentiated MSC after transplantation is another challenge, as collagen type II expression may be replaced by collagen type I, resulting in fibrocartilage production rather than the functional hyaline cartilage-like tissue [43, 46].

Moreover, these MSCs do not stop at the prehypertrophic stage, but continue to differentiate to become hypertrophic and thus less functional for cartilage repair, unlike prehypertrophic chondrocytes [32]. Indeed, functional chondrocytes at the outer layers of the articular cartilage (superficial, transitional, and radial) cease differentiation and keep their prehypertrophic phenotype. They continue to proliferate and repair the cartilage ECM. In contrast, in suspension culture, chondrocytes from the inner layer of the articular cartilage or from the center of the cartilaginous matrix stop proliferating and continue their differentiation to become enlarged (hypertrophic) cells, producing large amounts of collagen type X associated with ossification in later stages [47]. Cell-based therapy with surgical implantation of abundant amounts of MSC was found to be suitable for young patients and early stages of osteoarthritis, resulting in functional hyaline-like cartilage tissue repair. However, the unpredictable nature of the cartilage repair potential for other conditions remains an obstacle to the widespread use of MSC [34].

Validation of the *in vitro* modalities of chondrogenic differentiation of MSC remains a major problem, limiting the number of clinical studies in this field. To circumvent the disadvantages of MSC, new sources of MSC with a neural crest embryonic origin, especially from human orofacial tissues, may provide a highly serviceable alternative to MSC. These drawbacks have pushed researchers and clinicians to seek other cell sources for use in cartilage regeneration.

## 3. Neural Crest-Derived Cells as an Alternative to Mesoderm-Derived Cells for Cartilage Repair

### 3.1. Origin and Fate of Neural Crest-Derived Cells

During the embryonic stage called neurulation, neural crest cells are located between the neural plate and the neural ectoderm, forming a quasi “fourth germ layer” [48]. During gastrulation, cues engage their specification. At this stage, specific markers such as Snail2 (Slug), SOX9, SOX10, or FoxD3 are expressed. Neural crest cells remain in this location, dorsally, during neural tube formation. Thereafter, they lose their contact with the ectodermal cells surrounding them in a process called delamination. Then, during the epithelial-mesenchymal transition (EMT), they acquire a mesenchymal phenotype and migrate.

According to their initial location, neural crest cells colonize different areas, giving rise to specific cell derivatives. Several types of the neural crest tissue can be distinguished according to the initial segmentation affecting their destination, such as cranial neural crest.

The cranial neural crest cell contingent has remarkable pluripotency, as evidenced by the variety of tissues it generates. Cranial neural crest gives rise to both ectodermal-like cell types (e.g., neurons, Schwann cells, and melanocytes) and mesenchymal derivatives (e.g., osteoblasts and adipocytes). This understanding of “enlarged multipotency” was established *in vitro* by studying these cells at the clonal level [49, 50].

In adulthood, postmigratory neural crest cells typically acquire a differentiated phenotype (e.g., chondrocyte, osteoblast, fibroblast, and neural cell). However, most oral tissues contain stem cells that are neural crest-derived and can be induced to differentiate into neural or mesenchymal derivatives [51–54]. These cells can recreate different cell lineages *in vivo*. Thus, it was demonstrated that stem cells of the periodontal ligament have the capacity to reform *in vivo* various tissues necessary for the periodontal structure, namely the cementum and periodontal ligament [55]. When transplanted subcutaneously into immunocompromised mice, stem cells derived from the oral mucosa formed tumors consisting of two germ layer-derived tissues [52]. Furthermore, it was shown that cells derived from the dental follicle, when injected into blastocysts, could integrate into the internal mass of the embryo [56]. Consequently, stem cells have been isolated from many craniofacial structures (Figure 1(b)), including hair follicles of the facial skin (SKP) [57], dental pulp [58] of exfoliated deciduous teeth (SHED) [59], permanent teeth (DPSC), apical papilla (SCAP), dental follicle (DFSC) [60], periodontal ligament (PDLSC) [55], and gingival connective tissue (GSC) [61]. Stem cells were also recently found in the membrane of the maxillary sinus [62].

### 3.2. Neural Crest-Derived Chondrocytes: Nasal Chondrocytes

**Figure 2:**
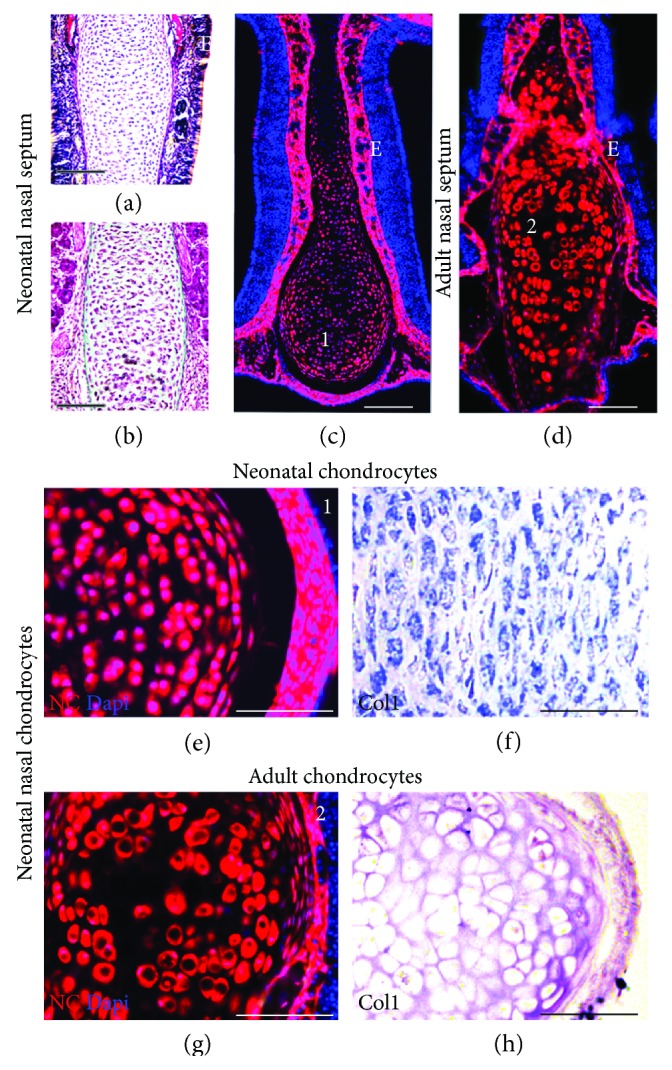
Neonatal and adult nasal septum. (a) Van Geison staining of chondrocytes of the neonatal mouse nasal septum showing developing chondrocytes (purple) with intercellular matrix (red) and collagen matrix (red). (b) Collagen network of the neonatal nasal septum shown in green by trichrome's stain. (c, d) Embryonic origin of neonatal and adult chondrocytes of the nasal septum cartilage from neural crest as expressed in transgenic mice expressing neural crest marker Pax3. (e, g) Transgenic mice expressing neural crest marker Pax3 confirm the neural crest-derived embryonic origin of the nasal chondrocytes: neonatal (e) and adult (g). (f, h) Histological sections of neonatal (e) and adult (h) nasal chondrocytes showing expression of Col1 as detected by hybridization in situ.

Among neural crest-derived cells, the ones used most often for cartilage repair are chondrocytes, notably nasal chondrocytes (Figure 2). Nasal cartilage provides an accessible source of chondrocytes when compared to the articular cartilage. *Ex vivo*, nasal chondrocytes can be expanded in culture dishes, where they acquire a fibroblastic phenotype with a high proliferative rate [16, 63], superior to that of chondrocytes derived from the articular or rib cartilage [64, 65]. Following amplification, the cells may be redifferentiated into chondrocytes to produce specific cartilage ECM proteins by using 3D culture techniques such as micropellets.

Owing to this accessibility and *ex vivo* expansion potential, they have been assessed *in vivo* for facial applications such as nasal septum reconstruction [66] as well as orthopedic applications. In the latter, engineered cartilage appeared as native-like cartilage in terms of its structural adaptiveness to mechanical loading [67]. Transplanted cells participated directly in neocartilage formation [68]. Moreover, these heterotopic grafts were characterized by the integration of environmental cues, enabled by the plasticity of the neural crest-derived chondrocytes [69].

Nasal chondrocytes have been tested in clinical trials for at least two different clinical indications. The first involved nasal restoration after excision of nonmelanoma skin cancer of the alar lobule [70]. Five patients were enrolled and treated with autologous cartilage constructs. These were composed of porcine-derived collagen type I and type III on which the expanded cells were seeded. One year later, histological analysis showed desirable remodeling of the grafted cartilage into fibromuscular fatty tissue corresponding to the native tissue of this site [70]. The other trial consisted of grafting a heterotopic cartilage construct for knee joint regeneration. In this case, the cartilage construct was like those used for nasal alar lobule repair. Ten patients were enrolled and followed for 24 months [71]. MRI was performed at 6 and 24 months and revealed a structure close to native cartilage. Moreover, patient comfort was improved 24 months after the surgery. These two trials confirmed the plasticity of cells derived from the nasal cartilage and their promising potential for cartilage repair.

Ear perichondrium tissue is an additional new source of stem/progenitor cells [72, 73]. These cells showed a higher proliferation rate than chondrocytes and produced more cartilage tissue weight than BM-MSC when transplanted *in vivo* [73]. They may also be used in elastic cartilage regeneration and plastic surgery [74].

Taken together, these results demonstrate the growing interest in using neural crest-derived chondrocytes for cartilage repair. When compared to articular chondrocytes, nasal chondrocytes displayed higher chondrogenic potential than articular chondrocytes in an articular cartilage defect in a goat model [69]. Interestingly, the Hox gene code that instructs the organization of the 3D body plan is modified during transplantation. Indeed, neural crest-derived cells are Hox-negative in their natural environment; however, when transplanted to a mesodermal environment (articular cartilage defect), these cells become Hox-positive. This conversion is driven by cell-cell contact, not by soluble factors [69]. Previous experiments on osteogenic progenitors have highlighted a similar change of Hox gene expression and also showed that the switch in expression is not reversible [75]. These data strongly support the idea that the integration of environmental cues is shared by diverse neural crest cell lines. However, owing to their very recent and still limited application in tissue engineering, the use of nasal chondrocytes needs further assessment, including in clinical studies, before they can be judged effective.

### 3.3. Neural Crest-Derived Mesenchymal Stem Cells from Oral Tissues

**Figure 3:**
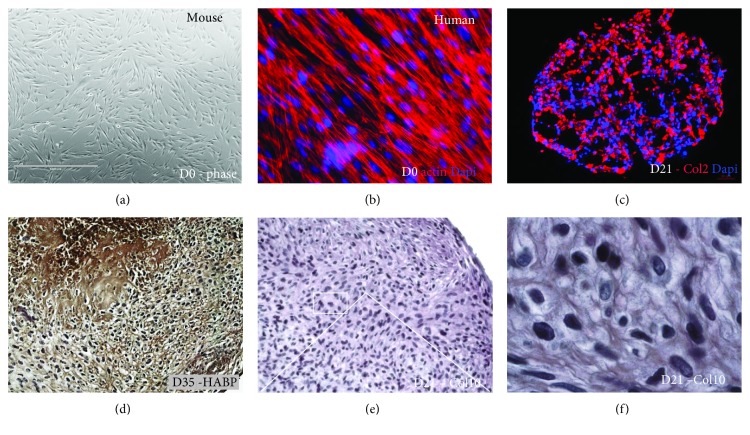
Chondrogenic differentiation potential of human and mouse NC-MSC: gingival stem cell example. (a) Mouse gingival stem cells (GSCs) were cultured for 21 days in suspension under suitable chondrogenic conditions. (b) Human GSCs were cultured for 21 days in suspension under suitable chondrogenic conditions. Immunostaining of actin and nuclei (Dapi) confirmed the initial fibroblastic phenotype. (c) Immunostaining of Col2 and cell nuclei (Dapi) in mouse GSC confirmed ECM cartilage synthesis in the chondrogenic pellet. (d) Immunostaining of hyaluronan-binding protein (HABP) in the GSC pellet after 35 days of chondrogenic differentiation. (e, f) Immunostaining of Col10 in the GSC pellet after 21 days of chondrogenic differentiation, showing the inner layers with low and high magnifications.

Adult oral tissues are a source of neural crest-derived mesenchymal stem cells (NC-MSCs). As such, they may be an alternative to nasal chondrocytes and mesodermal MSC in cartilage regeneration. Their isolation requires an invasive procedure, such as tooth extraction or dental pulp chamber trepanation. Gingival stem cell (GSC) isolation remains less invasive, as the tissue is harvested by a simple biopsy taken under local anesthesia without tooth loss or scar formation (Figure 3).

Several techniques have been described to isolate oral stem cells from a tissue sample. The most common is to grow the cells in an adherent plastic flask (monolayer). In this case, a heterogeneous population of cells migrates out of the biopsy. When a sufficient number of cells is obtained, the cells are detached and seeded at limiting dilution for selecting cells with clonogenic potential. To refine this method, a panel of surface markers was recently proposed. However, the markers used (CD29, CD44, CD73, CD90, CD105, CD106, CD146, and Stro-1) are unable to select stem cell populations [76]. Another method is to digest the sample with a range of enzymes to obtain a readily diluted population. This method can shorten the culture time and remove a trypsinization step. However, as with the previous method, the obtained population remains heterogeneous.

More recently, suspension culture methods have been developed. Initially used for cultivation of neural stem cells, this method has been transposed to NC-MSC, including those derived from oral tissues: DPSC, PDLSC, and GSC [77–80]. Apart from these oral sources, this culture method has been employed especially for SKP [81] and postmigratory NC-MSC present in bone marrow [82]. Under these conditions, the cells are cultured in nonadherent dishes in the serum-free medium supplemented with EGF and FGF. In this environment, the cells form neurospheres called microspheres that exhibit markers of neural crest and neural precursors (nestin, *β*3-tubulin) [83]. It has not been determined whether this method of growing selects a precursor or directs the cells to this lineage. Aggregating and maintaining these neurospheres require cell contacts through gap junctions (connexin 43) and cell-cell connections mediated by cadherin proteins [76, 84].

Apart from this ability to form neurospheres, NC-MSCs display properties which distinguish them from BM-MSC and AD-MSC. They express specific markers such as nestin and transcription factors such as Snail1, SOX10, or Twist1 [85]. Moreover, NC-MSCs show very interesting properties *in vitro*. For example, DPSCs show a higher proliferation rate [86] with a significantly lower doubling time than BM-MSC [87]. The proliferation rate of DPSC is higher than that of PDLSC [88], whereas GSCs grow faster and produce more CFU-F compared to PDLSC [89]. These data are important because clinical application in cartilaginous tissue regeneration requires large numbers of cells.

### 3.4. Neural Crest-Derived Oral Mesenchymal Stem Cells for Cartilage Repair

Several studies have investigated the chondrogenic potential of oral stem cells, including DPSC. It seems the natural chondrogenic potential of DPSC is absent or low [90]. However, one study showed that the side population (SP) of DPSC (isolated by flow cytometry according to the capacity of their DNA to not bind Hoechst 33342) had the ability to differentiate into chondrocytes *in vitro* [91]. Moreover, DPSC can differentiate if exposed to supplementary chemical cues such as TGF-*β*3 or if cocultured with costal chondrocytes [92, 93].

For both PDLSC and GSC, their chondrogenic differentiation potential has been demonstrated. For example, GSC grown as micromasses can differentiate into chondrocytes expressing specific markers such as SOX9 and COL2A1. Processes mimicking those involved in native chondrogenesis or reproduced in experiments using ESC or induced pluripotent stem cells (iPSC) were found to be involved [94]. Microscopic analysis showed collagen fibers parallel to each other and to the microsphere surface, as well as the presence of apoptotic cells during early differentiation, which are characteristics of native cartilage. Moreover, in these culture conditions, cells at the periphery differentiated into synovial cells expressing the specific marker cadherin-11 (CAD-11) [94]. When subjected to hypoxic conditions, these synovial cells differentiated into prehypertrophic chondrocytes with expression of specific markers such as COL10 (gene and protein), IHH, and MMP-13. However, although the medium was supplemented with *β*-glycerophosphate, no mineralization of the ECM was observed [94]. This contrasts with the behavior of BM-MSC, which produce mineralized matrix under these culture conditions.

Other studies have compared the chondrogenic potential of GSC to PDLSC *in vitro* and *in vivo*. Under *in vitro* conditions, PDLSCs exhibit higher chondrogenic capability compared to GSC, characterized by increased synthesis of ACAN and COL2A1 [89]. Increased osteogenic potential was also observed. However, when the culture medium was supplemented with proinflammatory molecules (TNF-*α* and IL-1*β*), the differences between GSC and PDLSC tended to lessen both *in vitro* and *in vivo* [89]. Although these proinflammatory conditions have been tested only in the osteogenic medium, these results support the potential immunomodulatory properties of GSC, giving them an advantage in the context of clinical applications. To improve the chondrogenic potential of these stem cells, differentiation protocols using several media and/or supports were tested. These included chitosan [95], hydrogel supporting alginate coupled to arginine-glycine-aspartic acid (RGD) [96], collagen type II, and hyaluronic acid mix, or a small intestine submucosal sheet [97, 98]. Protocols and major outcomes of these studies are described in Table 1.

Taken together, these results indicate that NC-MSCs have variable chondrogenic potential. Moreover, these data were obtained from *in vitro* experiments only. Thus, animal studies are necessary to assess the behavior of these cells in a cartilaginous environment.

In addition to the chondrogenic potential of NC-MSCs demonstrated in many studies, their anti-inflammatory properties are also promising for the treatment of cartilage pathology. Indeed, inflammation plays a key role in several articular pathologies such as osteoarthritis [99]. In this regard, GSCs are of particular interest because they possess anti-inflammatory properties in both the innate and adaptive immune systems, as demonstrated in several cell therapy protocols [100–102]. Based on these immunomodulatory properties, the effect of these cells was studied in a model of induced arthritis in mice. In this protocol, a group receiving an intravenous transfusion of GSC was compared to a group treated with dermal fibroblasts and a control group without treatment. The GSC-treated group showed a decrease in the severity of the disease compared to the other groups, including reduced synovial inflammation, pannus formation, and cartilage and bone destruction. These histological changes were associated with an increase of T-reg CD4+ CD39+ FoxP3+ cells that inhibit the synthesis of the proinflammatory cytokines IL-17 and IFN-*γ* [103].

Other strategies to obtain neural crest-derived cells look promising. For example, neural crest-like cells from human iPSC have been selected and amplified in the medium containing FGF2 and SB431542 (an inhibitor of the TGF-*β*/activin/nodal pathway). Under these conditions, these cells expressed the following membrane markers: PDGFR*α*, CD271, and CD73. Use of SB431542 and FGF2 also maintains the expression of SOX9 and CD271 while it decreases N-cadherin and SOX10 required for neural differentiation of neural crest-derived cells. These cells remained differentiated for at least 16 passages and required stimulation by TGF-*β* before mesenchymal condensation to activate chondrocyte differentiation. Microspheres first differentiated into chondrocytes and then, when transplanted subcutaneously into mice, they exhibited mineralization of the ECM. During this final stage of differentiation, murine cells but also human cells were involved in matrix mineralization, demonstrating that host cells directly participated in terminal differentiation [104]. In another study using a similar approach to obtain NC-MSC from iPSC, the cells were able to differentiate *in vitro* into chondrocytes. However, when these microspheres were transplanted in subchondral bone defects, they failed to restore the cartilage and bone, unlike BM-MSCs that were used as a positive control [105].

## 4. Conclusion

In cell therapy, the source of stem cells is crucial. The cellular properties linked to embryological origins have significant impacts for clinical applications. Compared to mesoderm-derived cells, neural crest-derived cells are easier to harvest, to amplify *in vitro*, and exhibit more stable differentiation into chondrocytes. Moreover, due to their intrinsic properties, they integrate cues from their environment with high plasticity. However, despite *in vitro*, *in vivo*, and some human trials, little is known about specific biological aspects of neural crest-derived cells. Up to now, most of the procedures used for selection, amplification, and differentiation of the cells have been copied from those used for mesodermal cells. Exploring the specificities of neural crest-derived cells for chondrocyte differentiation could improve the actual protocols and thereby contribute to the development of other sources of stem cells and better therapeutic outcomes. Moreover, neural crest-derived cells are heterogeneous in their chondrogenic potential and therefore require an appropriate choice of the cell source. Indeed, some sources exhibit limited or no potential for chondrogenic differentiation (i.e., DPSC). An additional limitation of the use of neural crest-derived cells is the donor variation affecting the quality of the cells harvested. Indeed, as for any autologous cell transplantation, the properties of cell amplification, differentiation potential, and response to environmental cues are partially dependent on the donor. This latter aspect remains a major challenge for cell therapy, but one that is being actively investigated.

## Figures and Tables

**(a):** 

Nasal chondrocytes						
Author	Cell types	Experiment type	Culture duration before implantation	Site of transplantation	Scaffold	Main results
Vinatier et al. (2009) [67]	Autologous nasal chondrocytes **Rabbit**	*In vitro* *In vivo*	4 weeks *in vitro* 6 weeks *in vivo*	Knee Rabbit	Si-HPMC hydrogel	Neocartilage: same histological aspect as healthy articular cartilage Analysis of col type II: hyaline-like cartilage

Fulco et al. (2014) [70]	Autologous nasal chondrocytes **Human**	Clinical trial	4 weeks *In vitro* 6 months Biopsy	Alar lobule Human	Collagen type I/III scaffold	Reconstructed tissues displayed fibromuscular fatty structures typical of the alar lobule stability and functionality of the grafts

Pelttari et al. (2014) [69]	Autologous nasal chondrocytes and articular chondrocytes **Goat**	*In vitro* *In vivo*	*In vitro*: 2 weeks *In vivo*: 5 weeks: cell plasticity; 3 and 6 months: preclinical effectiveness	Knee Goat	Collagen type I/III scaffold	*In vitro*: nasal chondrocytes: more efficient chondrogenic differentiation than articular chondrocytes (cloning and subcloning) *In vivo*: nasal chondrocytes gave a higher cartilage repair tissue quality than articular chondrocytes
	Nasal chondrocytes (*n* = 6) **Human**	*In vivo*	*In vitro*: 1 week *In vivo*: 5 weeks	Subcutaneous Nude mice	Collagen type I/III scaffold	Stability of Hox gene expression
	Nasal chondrocytes (septum) **Human**	*Clinical trial* *Phase 1*	4 weeks before implantation	Traumatic injury Knee Human	Collagen type I/III scaffold	(1) No systemic or local adverse events for follow-up patients to 18 months after implantation (2) Filling of the defect and no graft delamination, with strong reduction of subchondral bone edema 4 months after surgery

Mumme et al. (2016) [68]	Autologous nasal chondrocytes Autologous articular chondrocytes **Goat**	*In vivo*	*In vivo* 4-5 weeks cast 3-6 months	Knee Goat	Collagen type I and type III membrane (chondro-Gide)	Typical structures of articular cartilage with nasal chondrocytes. Efficient integration of the grafted tissues with the adjacent native cartilage and underlying subchondral bone with nasal chondrocytes No sign of osteoarthritis following the graft with nasal chondrocytes as compared to articular chondrocytes

Mumme et al. (2016) [71]	Autologous nasal chondrocytes **Human**	Clinical trial	24-month follow-up	Knee Human	Collagen type I/III scaffold	No adverse reactions Self-assessed clinical scores for pain, knee function, and quality of life were significantly improved Radiological assessments indicated variable degrees of defect filling and development of repair tissue approaching the composition of a native hyaline-like cartilage
	Nude mice	*In vivo* (tumorigenic tests)	8 weeks	Subcutaneous pockets	Collagen type I/III scaffold	Tumor-free tissues All explanted organs appeared macroscopically normal and no evidence of tumor formation was observed histologically

**(b):** 

NC-MSC						
Author	Cell types	Experiment type	Culture duration	Site of transplantation	Scaffold	Main results
Pierdormenico et al. (2006) [90]	DPSC (human) BM-MSC	*In vitro*	3-4 weeks	/	/	Failure of chondrogenic differentiation

Iohara et al. (2006) [91]	DPSC (SP) (porcin)	*In vitro*	45 days	/	/	Almost 30% of SP cells were converted into chondrocytes

Alge et al. (2010) [87]	DPSC (rat) BM-MSC (mouse)	*In vitro*	3 weeks	/	/	Both DPSC and BM-MSC achieved successful chondrogenic differentiation

Dai et al. (2012) [93]	DPSC+CC (human)	*In vitro* *In vivo*	/	Nude mice 8 weeks Subcutaneous	PGA	FGF9 enhanced chondrogenesis of DPSCs FGF9 inhibited hypertrophy and ossification in chondrodifferentiated DPSCs

Hsu et al. (2012) [95]	GF GSC (human)	*In vitro*	/	/	Chitosan	GSC isolation and culture on chitosan membranes increase their chondrogenic potential

Choi et al. (2013) [106]	PDLSC (human)	*In vitro*	14 days	/	/	TGF-*β*1 and BMP-6 stimulate chondrogenic differentiation of PDLSC If used together they may induce mineralization and hypertrophy

Moshaverinia et al. (2013) [96]	GSC PDLSC vs. BM-MSC (human)	*In vitro* *In vivo*	4 weeks	Nude mice Dorsal surface Subcutaneous	RGD-coupled alginate with high guluronic acid content	PDLSCs showed higher amounts of chondrogenesis and Sox9 and Coll II gene expression than BM-MSCs and GSCs *in vitro* and *in vivo*

Rizk et al. (2013) [92]	DPSC (human)	*In vitro* *In vivo*	/	Nude mice 12 weeks Lateral side	PLLA/PEG	TGF-*β*3 increases the chondrogenic potential of DPSC

Yang et al. (2013) [89]	PDLSC GSC (human)	*In vitro*	8 weeks	/	/	PDLSC had more chondrogenic differentiation potential than GSC

Ferré et al. (2014) [94]	GSC	*In vitro*	5 weeks	/	/	GSC in chondrogenic differentiation medium showed SOX9-dependent differentiation to both chondrocyte and synoviocyte lineages GSC in 3-week old medium: synovial cells peripheral positive to CAD-11

Nemeth et al. (2014) [107]	DPSC (mouse)	*In vitro*	/	10-21 days	PEG-GelMA-HA	Nanotopography and HA provide important cues for promoting chondrogenic differentiation of DPSCs

Yeh et al. (2014) [98]	Porcine Chondrocytes Human MSC: BM-MSC, AD-MSC, GF, PL-MSC	*In vitro* *In vivo*	3 weeks	NODScid mice Subcutaneous	CII-HA SIS	SIS scaffold more suitable for chondrogenic differentiation for all cell types. GF gave the best rate of chondrogenic differentiation on SIS scaffold

Umeda et al. (2015) [104]	IPSC (human) CD271+PDGFRa+CD73+ from the PAX3/SOX10/FOXD3-	*In vitro* *In vivo*	12 weeks	Dorsal skin NODScid/NSG mice	Gelfoam	The ectomesenchymal cells were expandable without loss of chondrogenic potential for at least 16 passages TGF-*β*3 promotes efficiently of these cells to form translucent cartilage particles, which were completely mineralized in 12 weeks in NODScid/NSG mice

Chijimatsu et al. (2017) [105]	Neural crest-derived mesenchymal stem cells from IPSC (iNCMSC)	*In vitro* *In vivo*	7 weeks	Osteochondral defect in euthymic nude rats	/	TGF-*β*3 alone in chondrogenic medium is not enough to ensure chondrogenic differentiation. BMP2 is required *In vivo*, chondrogenic particles failed to restore osteochondral defect contrary to BM-MSC
